# Some Recent Contributions to the Physiology of the Nervous System of Our Domestic Animals

**Published:** 1883-04

**Authors:** Charles L. Dana


### ART. X.—SOME RECENT CONTRIBUTIONS TO THE PHYSIOLOGY OF THE NERVOUS SYSTEM OF OUR DOMESTIC ANIMALS.

BY CHARLES L. DANA, A.M., M.D.,

AMONG somewhat over three hundred contributions to anatomy and physiology made during the past year, I find the authors distributed as follows: Germans 160, French 70, British 26, Italian 24, American 14, Scandinavian 13, Russian 6. Among the contributors to physiology America ranks as one of the last. It will be seen that nearly all of the work referred to below has been done by Germans; some of it by professors in veterinary schools. Though not of direct and and practical interest to veterinarians, I have thought that the accompanying brief notes might stimulate some interest in scientific study among the readers of the JOURNAL by showing how wide and important a field is covered by the comparative physiology of the domestic animals alone. John Hunter's fame, it is generally admitted, was largely due to his basing his surgery upon comparative physiology and anatomy.

*The Results of Section of the Vagus upon Sheep.*—Ellenberger has made some very interesting experiments to determine the effect of section of the pneumogastric nerves upon sheep. He found that after cutting the pneumogastric on one side only, no disturbance of heart, lungs, or stomach was observed. The general health was not impaired. Ten weeks after the section, the animals were killed. There appeared to be some thinning and atrophy of the muscular wall of the third and fourth stomach in the animal whose right vagus was cut, and a similar change in the first and second stomach of the animal whose left vagus was cut.

When both vagi were cut the animals died in from twelve to twenty-six hours, except in one case when life was prolonged for sixteen days. Death resulted in all cases from suffocation by stoppage of air passages.

There was constantly observed: complete paralysis of the œsophagus, partial paralysis of the first and second stomachs increased heart-beat up to 160 per minute, labored irregular and at first slower respiration, (twelve to sixteen per minute) and inability to regurgitate and chew the cud.

It appears that the vagus sends motor nerver fibres to the first and second stomachs, but that the third and fourth stomachs are innervated independently. The constant development of *tympanitis* as a result of paralysis of the vagus may have some practical significance.

*The Act of Swallowing in Dogs* is performed by muscles chiefly under the control of the vagus and glosso-pharyngeal nerves. Kronicker and Meltzer have shown that the glosso-pharyngeal nerve contains also inhibiting fibres, and that when these are stimulated the act of swallowing can not take place. When the glosso-pharyngeal of dogs is cut the oesophagus is thrown into a state of tonic spasm.

*Vertigo and Irregular Movements in Dogs* were produced by Bechterow upon destroying certain parts of the brain surface. The regions thus destroyed were not the so-called motor regions of the brain cortex, *i. e.*. those in the neighborhood of the crucial sulcus. (Fissure of Rolando in man), but rather behind these in the region of the distribution of the fibres from the thalamus opticus. The movements resembled those produced by injuring certain lower parts of the brain, *e. g.*, the Pons, the cerebral pedicles, and thalamus opticus. The animals after a time learned to overcome in part by voluntary movements the tendency to irregular movements.

*Destruction of the Cerebellum in Rabbits and Dogs.*—Baginsky succeeded in keeping alive four out of forty rabbits in whom he had removed small pieces of the cerebellar cortex (the vermis.) At first no change was noticed in the animals. After a few days, however, when a destructive process had extended deeper into the cerebellum, they showed uncertainty of gait, incoordination of movements and tremor. The appearance of the latter symptom to a certain extent corroborates Hughlings Jackson's and Spencer's view that the cerebellar-spinal system regulates the tonicity of muscles and movements in space. B's experiments also showed that when one part of the cerebellum is destroyed its function may be assumed by another part, as is the case with the cerebral lobes.

*A Compensating Development of the Cerebellum in Pigeons.*—Stefani extirpated both cerebral lobes of a pigeon. The animal survived and showed for three months the usual loss of spontaneity and intelligence. It then began to attempt to fly, to pick up food, etc. The animal was killed when a notable enlargement of the cerebellum was observed.

*The Seat of the " Music of the Hemispheres" in the Dog.*—H. Munk thinks that he has located the center of hearing in the cerebral cortex of the dog. And not only this, but he believes that different parts are devoted to the perception of different notes. This center for sound-sensations is in the convolution near the apex of the post-sylvian fissure. The posterior parts appreciate the low notes, while the more anterior portions appreciate the highèr notes.

Goltz and Couty, experimenting upon dogs and apes, combat the somewhat radical views of H. Munk and Ferrier.

*The Protective Function for Animals of the Semicircular Canals of the Ear.*—Necessity compels most of the higher animals, but especially wild animals to be constantly on the alert, and the sense of hearing is of especial value in self-protection. Dr. P. McBride endeavors to prove the theory (which is not entirely new) that the semi-circular canals have an important function in this matter. Sudden sounds conveyed to the internal ear excite the nerve fibres. These reflexly cause a sudden turning of the head in the direction of the noise. This action is seen when a deer is suddenly starled by a whistle.

The other function of the semi-circular canals relates probable to the sense of space, Stefani has recently asserted that that the cells of Purkinje in the cerebellum were in close relation with the semi-circular canals, so that when the latter are destroyed the former elements atrophy.

*Seat of " Good Nature" and Intelligence in Dogs.*—Goltz, by experiments upon dogs, shows that extensive and profound destruction of both vertex convolutions causes not only diminished intelligence, but also a remarkable change in their disposition. Harmless and good-natured dogs, after this operation, become surly, quarrelsome, and violent.

If the occipetal lobes are damaged the animals preserve their good-tempered character, but the intelligence is more seriously impaired.

*Consciousness of the Spinal Cord.*—Oehl has made experiments upon frogs and eels. He considers, upon nearly the same grounds as Pflueger and Hammond, that the spinal cord in the inferior animals possesses a kind of consciousness and intelligence. Even if this view, which is still a mere hypothesis, could be demonstrated as regards cold-bloods animals it might still fail to apply to the higher animals. Since the spinal cord occupies comparatively a more and more subordinate place in relation to conscious intelligence as animals ascend in the scale.

